# Prevalence and Spectrum of Germline *BRCA1* and *BRCA2* Variants of Uncertain Significance in Breast/Ovarian Cancer: Mysterious Signals From the Genome

**DOI:** 10.3389/fonc.2021.682445

**Published:** 2021-06-11

**Authors:** Daniele Fanale, Alessia Fiorino, Lorena Incorvaia, Alessandra Dimino, Clarissa Filorizzo, Marco Bono, Daniela Cancelliere, Valentina Calò, Chiara Brando, Lidia Rita Corsini, Roberta Sciacchitano, Luigi Magrin, Alessia Pivetti, Erika Pedone, Giorgio Madonia, Alessandra Cucinella, Giuseppe Badalamenti, Antonio Russo, Viviana Bazan

**Affiliations:** ^1^ Department of Surgical, Oncological and Oral Sciences, Section of Medical Oncology, University of Palermo, Palermo, Italy; ^2^ Department of Biomedicine, Neuroscience and Advanced Diagnostics (Bi.N.D.), Section of Medical Oncology, University of Palermo, Palermo, Italy

**Keywords:** *BRCA1*, *BRCA2*, breast cancer, genetic testing, ovarian cancer, variants of uncertain significance (VUS)

## Abstract

About 10–20% of breast/ovarian (BC/OC) cancer patients undergoing germline *BRCA1/2* genetic testing have been shown to harbor Variants of Uncertain Significance (VUSs). Since little is known about the prevalence of germline *BRCA1/2* VUS in Southern Italy, our study aimed at describing the spectrum of these variants detected in BC/OC patients in order to improve the identification of potentially high-risk *BRCA* variants helpful in patient clinical management. Eight hundred and seventy-four BC or OC patients, enrolled from October 2016 to December 2020 at the “Sicilian Regional Center for the Prevention, Diagnosis and Treatment of Rare and Heredo-Familial Tumors” of University Hospital Policlinico “P. Giaccone” of Palermo, were genetically tested for germline *BRCA1/2* variants through Next-Generation Sequencing analysis. The mutational screening showed that 639 (73.1%) out of 874 patients were *BRCA*-*w.t.*, whereas 67 (7.7%) were carriers of germline *BRCA1/2* VUSs, and 168 (19.2%) harbored germline *BRCA1/2* pathogenic/likely pathogenic variants. Our analysis revealed the presence of 59 different VUSs detected in 67 patients, 46 of which were affected by BC and 21 by OC. Twenty-one (35.6%) out of 59 variants were located on *BRCA1* gene, whereas 38 (64.4%) on *BRCA2*. We detected six alterations in *BRCA1* and two in *BRCA2* with unclear interpretation of clinical significance. Familial anamnesis of a patient harboring the *BRCA1*-c.3367G>T suggests for this variant a potential of pathogenicity, therefore it should be carefully investigated. Understanding clinical significance of germline *BRCA1/2* VUS could improve, in future, the identification of potentially high-risk variants useful for clinical management of BC or OC patients and family members.

## Introduction

Since the discovery of *BRCA1* and *BRCA2* genes, genetic testing requests have been steadily increasing. Inherited Pathogenic Variants (PVs) or Likely Pathogenic Variants (LPVs) detected in these major susceptibility genes have been shown to be involved in the Hereditary Breast and Ovarian Cancer syndrome (HBOC) (1–4). However, these sequence variants confer in carriers an increased lifetime risk also to develop other tumors such as pancreatic carcinoma (5, 6), prostate cancer (7–9), and melanoma (10).

As regards the meaning of variants, the Italian Association of Medical Oncology (AIOM) adopts the classification criteria proposed by the Evidence-based Network for the Interpretation of Germline Mutant Alleles (ENIGMA) consortium (https://enigmaconsortium.org/), according to the International Agency for Research on Cancer (IARC) recommendations (11), considering a classification system of variants into five classes: Benign (class I), Likely Benign (class II), Variant of Uncertain Significance (VUS, class III), Likely Pathogenic (class IV), and Pathogenic (class V) (12). Thanks to Next-Generation Sequencing (NGS) technologies, novel variants defined as VUSs have been shown to be harbored by 10–20% of patients undergoing *BRCA1/2* genetic screening (13). A VUS is a nucleotide sequence alteration with unknown consequences on the possible loss of function of the gene product or on the potential risk of causing disease. Consequently, the clinical significance remains unclear leading to a difficult clinical management by the oncologist and not easy explanation to the patient, since a VUS exhibits a probability of being reclassified as pathogenic between 5 and 94.9% (14).

Most of VUSs could have no effect on the *BRCA1/2* tumor suppressor function. Some of these variants behave as low-penetrance gene mutations and should not be managed as highly penetrant alterations (15). However, many of these may be crucial in the inheritance of high-risk neoplasms, and therefore, their identification in family members could be essential. As consequence, in case of VUS identification in the genome of a proband, the clinical decisions concerning the risk reduction and prevention strategies depend on family history and other risk factors, pending a reclassification of the variant. Generally, it has been demonstrated that most of VUS will be subsequently reclassified as class I benign variants (16, 17). The aim of the *BRCA Challenge project* and the *BRCA Exchange* database is to minimize differences in clinical interpretations of VUS between different laboratories, both worldwide and in national territory (18–20). Nowadays, *in silico* and experimental approaches are adopted for the classification of new and unclear sequence variants in order to improve the clinical management of *BRCA1/2* VUS carriers. Data regarding family history of cancer are integrated as co-segregation with disease and co-occurrence with known PVs/LPVs into computational models in order to assess the probability that a VUS is a cause of disease (21).

To date, the knowledge about the prevalence of *BRCA1/2* VUS in BC or OC patients belonging to some regions of Southern Italy such as Sicily is poor. Based on a Breast and Ovarian Cancer BRCA System database harvested at the University Hospital Policlinico “P. Giaccone” of Palermo, the aim of this retrospective investigation was to describe the typology and gene location of germline variants of unknown significance detected in *BRCA1* and *BRCA2* coding sequences and splicing sites of BC or OC patients in order to investigate the prevalence and spectrum of these inherited genetic variants observed in Southern Italy. Furthermore, the analysis of all molecular and clinical data of BC or OC patients could favor the identification of potentially high-risk susceptibility variants distributed in Sicilian population, contributing to the future reclassification of these variants with unclear clinical significance. Therefore, this work may provide information which, in the future, could be helpful in the clinical management of BC/OC patients harboring VUSs.

## Patients and Methods

### Study Population

A retrospective collection of clinical and molecular data from 874 unrelated BC or OC patients was carried out from October 2016 to December 2020 at the “Sicilian Regional Center for the Prevention, Diagnosis and Treatment of Rare and Heredo-Familial Tumors” of the Section of Medical Oncology of University Hospital Policlinico “P. Giaccone” of Palermo. Eight hundred seventy-four probands, 531 of which were with BC and 343 with OC, have been subjected to *BRCA* genetic testing according to specific susceptibility criteria based on a strong family and personal history of BC and/or OC. BC and/or OC patients with at least two other family members affected by HBOC-associated tumors are considered individuals with a strong family history. The personal and family anamnesis of patients was acquired during oncogenetic counseling performed by a multidisciplinary group of specialists which included an oncologist, a geneticist, and a psychologist. All patients have provided a signed informed consent. All information regarding personal and familial history of cancer, family geographical origin, age at diagnosis, histological tumor subtype, molecular phenotype and disease stages (I–IV) was anonymously recorded. The study (Protocol “G-Land 2017”) was approved by the ethical committee (Comitato Etico Palermo 1; approval number: 0103-2017) of the University-affiliated Hospital A.O.U.P. ‘P. Giaccone’ of Palermo. Data concerning the histological type and cancer diagnosis were provided by medical pathology reports in diagnostic core biopsies or tumor resections.

All patients were screened for germline *BRCA1/2* genetic testing based on probability rate of carrying *BRCA1/2* variants calculated by means of BRCAPRO genetic risk prediction model (22, 23) and according to the criteria established by guidelines of the Italian Association of Medical Oncology (AIOM) (24).

The genetic analysis result has been considered informative when a PV/LPV was identified or non-informative if no PV/LPV was detected, but it was not excluded that it could be present, or if a VUS, belonging to class III, was found (15, 25). A genetic testing will be positive in case of identification of inherited PVs/LPVs, but negative only in case of lack of identification of a known PV/LPV in a family member.

### Sample Collection and Next-Generation Sequencing Analysis

Peripheral blood samples were harvested at diagnosis from BC or OC patients through a vacutainer syringe containing EDTA. Genomic DNA was isolated using the DNeasy^®^ Blood Kit (QIAGEN, Hilden, Germany). After the extraction phase, DNA has been quantified by Qubit^®^3.0 fluorometer (Thermofisher Scientific, Waltham, MA, USA) and its quality has been assessed through the use of 2100 Bioanalyzer (Agilent Technologies, Santa Clara, CA).

The genetic analysis for *BRCA1/2* was performed as previously described (4, 26).

Sequencing analysis was performed using Ion 520 Chip (Thermofisher Scientific, Waltham, MA, USA) and Ion Torrent S5 (Thermofisher Scientific, Waltham, MA, USA) NGS platform. Obtained data were analyzed using both Amplicon Suite (SmartSeq s.r.l.) and Ion Reporter Software v.5.12 (Thermofisher Scientific, Waltham, MA, USA). NGS data analysis was performed with the standardization of sequencing coverage depth in order to minimize the probability of false positive and negative results in clinical practice, considering a minimum coverage of 500× to each sample.

### Sanger Sequencing Analysis

Sanger sequencing analysis was used to confirm the BRCA1/2 VUS identified by NGS analysis. We used the SeqStudio analyzer (Thermofisher Scientific, Waltham, MA, USA) and BigDye Therminator 3.1 Cycle Sequencing Kit (Life Technologies, Carlsbad, CA, USA), according to manufacturer’s protocols, as previously described (26).

### Genetic Variant Classification

Identified *BRCA* variants have been classified according to the criteria developed by the Evidence-based Network for the Interpretation of Germline Mutant Alleles (ENIGMA) consortium (https://enigmaconsortium.org/) and IARC recommendations (11), using a system of classification in five classes: benign (class I), likely benign (class II), variant of uncertain significance (VUS, class III), likely pathogenic (class IV), and pathogenic (class V). After variant meaning understanding, available databases, such as ClinVar, VarSome and Priors V2.0 Software packages, were our weapon to perform an *in silico* analysis in order to investigate the molecular and clinical meaning of an identified variant with unclear properties. Algorithms developed to predict the effect of missense changes on protein structure and function (PolyPhen-2, SIFT, Align-GVGD) have been used.

The localization of the variants on *BRCA1* and *BRCA2* genes was obtained and graphically represented using the informatic tool Mutation Mapper-cBioPortal for Cancer Genomics (27, 28). The identified *BRCA1/2* VUS was named according to the systematic nomenclature of the recommendations for the description of sequence variants established by the Human Genome Variation Society (HGVS) with authorization by the HGVS, Human Variome Project (HVP), and the Human Genome Organization (HUGO) (29).

## Results

### Detection of Germline *BRCA1/2* VUS in BC or OC Patients

Eight hundred seventy-four patients with BC or OC, enrolled from October 2016 to December 2020 at our institute, who met the criteria concerning personal and family history of cancer recommended by the AIOM national guidelines, were genetically tested for germline *BRCA1/2* variants. Among recruited 874 probands, 531 were BC patients and 343 were OC women.

The mutational screening of the examined study cohort showed that 639 (73.1%) out of 874 patients harbored germline *BRCA1/2* benign/likely benign variants (*BRCA*-*w*.*t*.), whereas 67 (7.7%) probands were carriers of germline *BRCA1/2* VUS (class III), and 168 (19.2%) subjects carried a germline *BRCA1/2* PV/LPV (*BRCA*-positive) (**Supplementary Figure 1**).

The genetic analysis revealed the presence of 59 different VUS detected in 67 patients, 46 of which had BC (68.7%) and 21 were women affected by OC (31.3%). The median age at diagnosis of analyzed BC or OC patients was 45 years (range 22–75 years) and 51 years (range 28–78 years), respectively. In particular, most of BC patients had an invasive ductal carcinoma (31/46 probands; 67.4%), a luminal A molecular phenotype (15/46; 32.6%), and more than one relative with *BRCA*-related tumor (22/46; 47.8%). Furthermore, 18 (39%) out 46 probands harbored early onset BCs (before age 40 year), 16 of which were hormone-dependent tumors and 2 triple-negative BCs (**Table 1**). On the other side, most of OC patients had monolateral ovarian carcinoma with high-grade serous carcinoma (HGSC) histological subtype (16/21 women; 76.2%) (**Table 1**).

**Table 1:** Clinico-pathological features of germline *BRCA1/2* VUS carriers affected by BC or OC.

BC (n = 46)	No. Patients (%)
**Sex**	
Female	44
Male	2
**Age at Diagnosis (years)**	
**Median (range)**	45 (22-75)
≤40	18 (39%)
41–50	15 (32.6%)
51–60	11 (24%)
≥ 60	2 (4.4%)
**Histology**	
Ductal *in situ*	6 (13%)
Invasive ductal	31(67.4%)
Invasive lobular	5 (10.9%)
Others	4 (8.7%)
**Molecular phenotype**	
Luminal A	15 (32.6%)
Luminal B/HER2 -	13 (28.3%)
Luminal B/HER2 +	4 (8.7%)
HER2 + (non-luminal)	1 (2.1%)
Triple negative	13(28.3%)
**No. of family members with cancer history**	
0	20 (43.5%)
1	4 (8.7)
>1	22 (47.8%)
**Type of surgery**	
Mastectomy	15 (32.6%)
Breast conserving therapy	26(56.5%)
Unknown	5 (10.9%)
**OC (n = 21)**	
**Age at Diagnosis (years)**	
**Median (range)**	51 (28-78)
≤ 40	1 (4.8%)
41–50	8 (38.1%)
51–60	8 (38.1%)
≥ 60	4 (19.0%)
**Cancer site**	
Ovarian carcinoma	16 (76.2%)
Bilateral Ovarian Carcinoma	5 (23.8%)
Fallopian tube carcinoma	0 (0%)
Primary peritoneal carcinoma	0 (0%)
**FIGO stages**	
Stage I	4 (19%)
Stage II	2 (9.5%)
Stage III	7 (33.4%)
Stage IV	0 (0%)
Unknown	8 (38.1%)
**Histological Subtype**	
HGSC	16 (76.2%)
Clear cell	1 (4.8%)
Endometrioid	3 (14.2%)
LGSC	0 (0%)
Papillary	1 (4.8%)
**N. of family members with cancer history**	
0	4 (19%)
1	2 (9.5%)
>1	9 (42.8%)
Unknown	6 (28.7%)

HGSC, high-grade serous carcinoma; LGSC, low-grade serous carcinoma.

Considering a distinction for pathology, among the 46 BC patients, 18 different variants have been identified in *BRCA1* gene and 33 in *BRCA2* gene. Among the 21 OC women, eight harbored *BRCA1* VUS and 13 *BRCA2* VUS.

Twenty-five (37.3%) out 67 probands, 17 of which were affected by BC and eight by OC, harbored VUS in *BRCA1* gene; 37 (55.2%) subjects, 24 of which were with BC and 13 with OC in *BRCA2* gene, whereas four (6.0%) BC women carried simultaneously two VUS in *BRCA2*, and one (1.5%) BC patient had VUS both in *BRCA1* and *BRCA2*. Twenty-one (35.6%) out of 59 different variants were located on *BRCA1* gene, whereas 38 (64.4%) have been identified on *BRCA2* gene (**Tables 2** and **3**).

**Table 2:** *BRCA1* gene variants of unclear significance harboured by patients with breast and ovarian cancers.

*BRCA1* VUS										
Nucleotide change HGVS nomenclature	Amino acid change	Type of VUS	ClinVar classification	ENIGMA/VarSome	PolyPhen-2/SIFT	HCI Prior/Align-GVGD^a^	BC patients	OC patients	ExAC/GnomAD^b^	pCR
**c.3367G>T**	p.Asp1123Tyr	MISSENSE	CIP	NYR/VUS	Light/Damaging	0.02/C0	2	\	0.00002/0.00001	OCCR
**c.889A>C**	p.Met297Leu	MISSENSE	CIP	NYR/VUS	Light/Tolerated	0.02/C0	1	\	\	\
**c.2417C>C**	p.Ala806Gly	\	NF	No data	Light/-	0.02/C0	1	\	\	OCCR
**c.81-12dupC**	\	IVS	NF	No data	\	\	1	\	\	\
**c.301+6T>C**	\	IVS	VUS	NYR/VUS	\	0.34 /\	1	\	0.00002/0.00001	BCCR1
**c.4063_4065delAAT**	p.Asn1355del	In-frame DEL	CIP	NYR/VUS	\	\	1	\	\	\
**c.4460A>C**	p.Lys1487Arg	MISSENSE	VUS	NYR/VUS	Light/Tolerated-Damaging	0.02/C0	1	\	\	BCCR2
**c.1881C>C**	p.Val627=	synonymous	CIP	NYR/LBV	\	0.02 /\	1	1	-/0.00003	OCCR
c.2447A>C*	p.His816Arg	MISSENSE	CIP	NYR/VUS	Light/Tolerated	0.02/C0	1	\	0.00001/0.00002	OCCR
**c.3952A>C**	p.Ile1318Val	MISSENSE	VUS	NYR/VUS	Light/Tolerated	0.02/C0	1	\	\	OCCR
**c.742A>C**	p.Thr248Pro	MISSENSE	CIP	NYR/VUS	Light/Tolerated	0.02/C0	1	\	\	\
**c.4185+8_4185+8delG**	\	IVS	NF	NF/VUS	\	\	1	\	\	\
**c.4054G>A**	p.Glu1352Lys	MISSENSE	VUS	NYR/VUS	Light/Damaging	0.02/C0	1	\	0.00004/0.00002	OCCR
**c.4739C>T**	p.Ser1580Phe	MISSENSE	VUS	NYR/VUS	Light/Damaging	0.02/C15	1	\	\	BCCR2
**c.4009G>C**	p.Asp1337His	MISSENSE	VUS	NYR/VUS	Light/Tolerated	0.02/C0	1	\	\	OCCR
**c.2218G>C**	p.Val740Leu	MISSENSE	VUS	NYR/VUS	Light/Tolerated	0.02/C0	1	\	\	OCCR
**c.4096+3A>C**	\	IVS	VUS	VUS/LPV	\	0.97 /\	1	1	\	\
**c.4963T>C**	p.Ser1655Ala	MISSENSE	Not provided	NYR/LPV	Light/Damaging	0.03/C0	\	3	\	\
**c.4543G>A**	p.Gly1515Arg	\	NF	NF/VUS	Light/Tolerated	0.02/C0	\	1	\	BCCR2
**c.1007C>T**	p.Thr336Ile	\	NF	NF/VUS	Light/Damaging	0.02/C0	\	1	\	\
**c.1705A>C**	p.Asn569Asp	MISSENSE	VUS	NYR/VUS	Light/Tolerated	0.02/C0	\	1	\	OCCR

*This BRCA1 variant is simultaneously present together with the BRCA2 VUS c.8262T>G (reported in **Table 3**) in one of probands with BC. Novel variants are reported in bold character.

a The Align-GVGD program predicts where the variants in BRCA1 and BRCA2 genes fall in a spectrum ranging from enriched deleterious to enriched neutral. The prediction classes form a spectrum (C0, C15, C25, C35, C45, C55, C65) with C65 most likely to interfere with protein function and C0 least likely. The HCI Prior database, based on Align-GVGD scores, defines four classes of probability of pathogenicity: C0 = 0.03; C15–C25 = 0.29; C35–C55 = 0.66; C65 = 0.81.

b The Exome Aggregation Consortium (ExAC) and Genome Aggregation Database (gnomAD) aggregate both exome and genome sequencing data from a wide variety of large-scale sequencing projects, by providing values of allelic frequency.

BC, breast cancer; BCCR, Breast Cancer Cluster Region; CIP, Conflicting Interpretations of Pathogenicity; DEL, Deletion; HGVS, Human Genome Variant Society; IVS, intronic variants; LBV, likely benign variant; LPV, likely pathogenic variant; NF, not found; NYR, not yet reviewed; OC, ovarian cancer; OCCR, Ovarian Cancer Cluster Region; pCR, putative Cluster Region (defined by Rebbeck et al.); VUS, Variant Of Uncertain Significance.

**Table 3:** *BRCA2* gene variants of unclear significance harbored by patients with breast and ovarian cancers.

*BRCA2* VUS										
Nucleotide change HGVS nomenclature	Amino acid change	Type of VUS	ClinVar classification	ENIGMA/VarSome	PolyPhen-2/SIFT	HCI Prior/Align-GVGD^a^	BC patients	OC patients	ExAC/GnomAD^b^	pCR
c.9839C>A**	p.Pro3280His	MISSENSE	CIP	NYR/VUS	Light/Damaging	0.81/C65	3	\	0.00002/0.00002	\
c.1769T>G**	p.Phe590Cys	MISSENSE	CIP	NYR/VUS	Light/Damaging	0.02/C65	3	\	0.00002/0.00001	BCCR1’
**c.8299C>T**	p.Pro2767Ser	MISSENSE	CIP	NYR/VUS	Light/Damaging	0.81/C65	2	\		BCCR2
**c.8393C>G**	p.Pro2798Arg	MISSENSE	VUS	NYR/VUS	Light/Damaging	0.81/C65	2	\		BCCR2
**c.3517A>T**	p.Ile1173Phe	MISSENSE	VUS	NYR/VUS	Light/Tolerated	0.02/C0	1	1		OCCR1
c.4960T>G***	p.Cys1654Gly	MISSENSE	VUS	NYR/VUS	Light/Damaging	0.02/C0	1	\		OCCR1
**c.3762G>T**	p.Glu1254Asp	MISSENSE	VUS	NYR/VUS	Light/Damaging	0.02/C0	1	\	0.00001/0.00000	OCCR1
**c.68-7delT**	\	IVS	CIP	NYR/VUS	\	\	1	\		BCCR1
**c.9838C>T**	p.Pro3280Ser	MISSENSE	CIP	NYR/VUS	Light/Damaging	0.81/C65	1	\	0.00002/0.00002	\
**c.3073A>G**	p.Lys1025Glu	MISSENSE	CIP	NYR/VUS	Light/Damaging	0.02/C0	1	\	0.00004/0.00005	\
**c.5267T>A**	p.Val1756Glu	MISSENSE	VUS	NYR/VUS	Light/Damaging	0.02/C0	1	\	0.00001/0.00000	OCCR1
**c.298A>G**	p.Lys100Glu	MISSENSE	VUS	NYR/VUS	Light/Damaging	0.02/C0	1	\		BCCR1
**c.8091C>A**	p.Ser2697Arg	MISSENSE	VUS	NYR/VUS	Light/Damaging	0.29/C15	1	\		BCCR2
**c.8632+2T>C**	\	IVS	NF	NYR/PV	\	0.97 /\	1	\		BCCR2
**c.5428G>A**	p.Val1810Ile	MISSENSE	CIP	NYR/VUS	Light/Damaging	0.02/C0	1	\	0.00002/0.00002	OCCR1
**c.5423T>C**	p.Ile1808Thr	MISSENSE	CIP	NYR/VUS	Light/Tolerated	0.02/C0	1	\	0.00002/0.00002	OCCR1
c.8262T>G*	p.His2754Gln	MISSENSE	VUS	NYR/VUS	Light/Tolerated	0.03/C0	1	\		BCCR2
**c.5663A>G**	p.Lys1888Arg	MISSENSE	CIP	NYR/VUS	Light/Tolerated	0.02/C0	1	\	0.00002/0.00001	OCCR1
c.4516T>C***	p.Phe1506Leu	MISSENSE	CIP	NYR/VUS	Light/Tolerated	0.02/C0	1	\		OCCR1
**c.8419T>C**	p.Ser2807Pro	MISSENSE	VUS	NYR/VUS	Light/Tolerated	0.81/C65	1	1		BCCR2
**c.1700C>T**	p.Thr567Ile	MISSENSE	VUS	NYR/VUS	Light/Tolerated	0.02/C0	1	\	0.00001/0.00000	BCCR1’
**c.464G>C**	p.Arg155Thr	MISSENSE	VUS	NYR/VUS	Light/Damaging	0.02/C0	1	\	0.00001/-	BCCR1
**c.9581C>A**	p.Pro3194Gln	MISSENSE	CIP	NYR/VUS	Light/Damaging	0.02/C0	1	\	0.00001/0.00003	\
**c.9076C>G**	p.Gln3026Glu	MISSENSE	CIP	NYR/VUS	Light/Damaging	0.03/C0	1	\	0.00002/0.00001	\
**c.3509C>T**	p.Ala1170Val	MISSENSE	CIP	NYR/VUS	Light/Tolerated	0.02/C0	1	\	0.00001/0.00002	OCCR1
**c.7954G>A**	p.Val2652Met	MISSENSE	VUS	NYR/VUS	Light/Damaging	0.29/C15	1	\	-/0.00000	BCCR2
**c.5897A>G**	p.His1966Arg	MISSENSE	CIP	NYR/VUS	Light/Damaging	0.02/C0	1	\	0.00002/0.00003	\
**c.4928T>C**	p.Val1643Ala	MISSENSE	CIP	NYR/LBV	Light/Damaging	0.02/C0	\	1	0.00005/0.00003	OCCR1
**c.9006A>T**	p.Glu3002Asp	MISSENSE	CIP	NYR/VUS	Light/Damaging	0.66/C35	\	1		\
**c.2981C>T**	p.Ala994Val	MISSENSE	VUS	NYR/VUS	Light/Tolerated	0.02/C0	\	1	0.00001/0.00000	\
**c.10150C>G**	p.Arg3384Gly	MISSENSE	VUS	NYR/VUS	Light/Tolerated	\	\	1		\
**c.4550A>G**	p.Lys1517Arg	MISSENSE	VUS	NYR/VUS	Light/Tolerated	0.02/C0	\	1		OCCR1
**c.3536G>A**	p.Ser1179Asn	MISSENSE	Not provided	NYR/VUS	Light/Tolerated	0.02/C0	\	1	-/0.00000	OCCR1
**c.4277C>A**	p.Thr1426Lys	MISSENSE	VUS	NYR/VUS	Light/Tolerated	0.02/C0	\	1		OCCR1
**c.5492T>C**	p.Ile1831Thr	MISSENSE	VUS	NYR/VUS	Light/Tolerated	0.02/C0	\	1	0.00001/0.00000	OCCR1
**c.9477C>A**	p.Phe3159Leu	MISSENSE	CIP	NYR/VUS	Light/Damaging	0.03/C0	\	1	-/0.00000	\
**c.5669T>C**	p.Met1890Thr	MISSENSE	VUS	NYR/VUS	Light/Damaging	0.02/C0	\	1	-/0.00000	OCCR1
**c.831T>G**	p.Asn277Lys	MISSENSE	CIP	NYR/VUS	Light/Damaging	0.02/C0	\	1	0.00006/0.00003	BCCR1’

*This BRCA2 VUS is simultaneously present together with the BRCA1 variant c.2447A>G (reported in **Table 2**) in one of probands with BC.

**These two BRCA2 variants are simultaneously present in three different probands with BC.

***These two BRCA2 variants are simultaneously present in one proband with BC.

a The Align-GVGD program predicts where the variants in BRCA1 and BRCA2 genes fall in a spectrum ranging from enriched deleterious to enriched neutral. The prediction classes form a spectrum (C0, C15, C25, C35, C45, C55, C65) with C65 most likely to interfere with protein function and C0 least likely. The HCI Prior database, based on Align-GVGD scores, defines four classes of probability of pathogenicity: C0 = 0.03; C15–C25 = 0.29; C35–C55 = 0.66; C65 = 0.81.

b The Exome Aggregation Consortium (ExAC) and Genome Aggregation Database (gnomAD) aggregate both exome and genome sequencing data from a wide variety of large-scale sequencing projects, by providing values of allelic frequency.

BC, breast cancer; BCCR, Breast Cancer Cluster Region; CIP, Conflicting Interpretations of Pathogenicity; HGVS, Human Genome Variant Society; IVS, intronic variant; LBV, likely benign variant; NF, not found; NYR, not yet reviewed; OC, ovarian cancer; OCCR, Ovarian Cancer Cluster Region; pCR, putative Cluster Region (defined by Rebbeck et al.); PV, pathogenic variant; VUS, Variant of Uncertain Significance.

**Figure 1:**
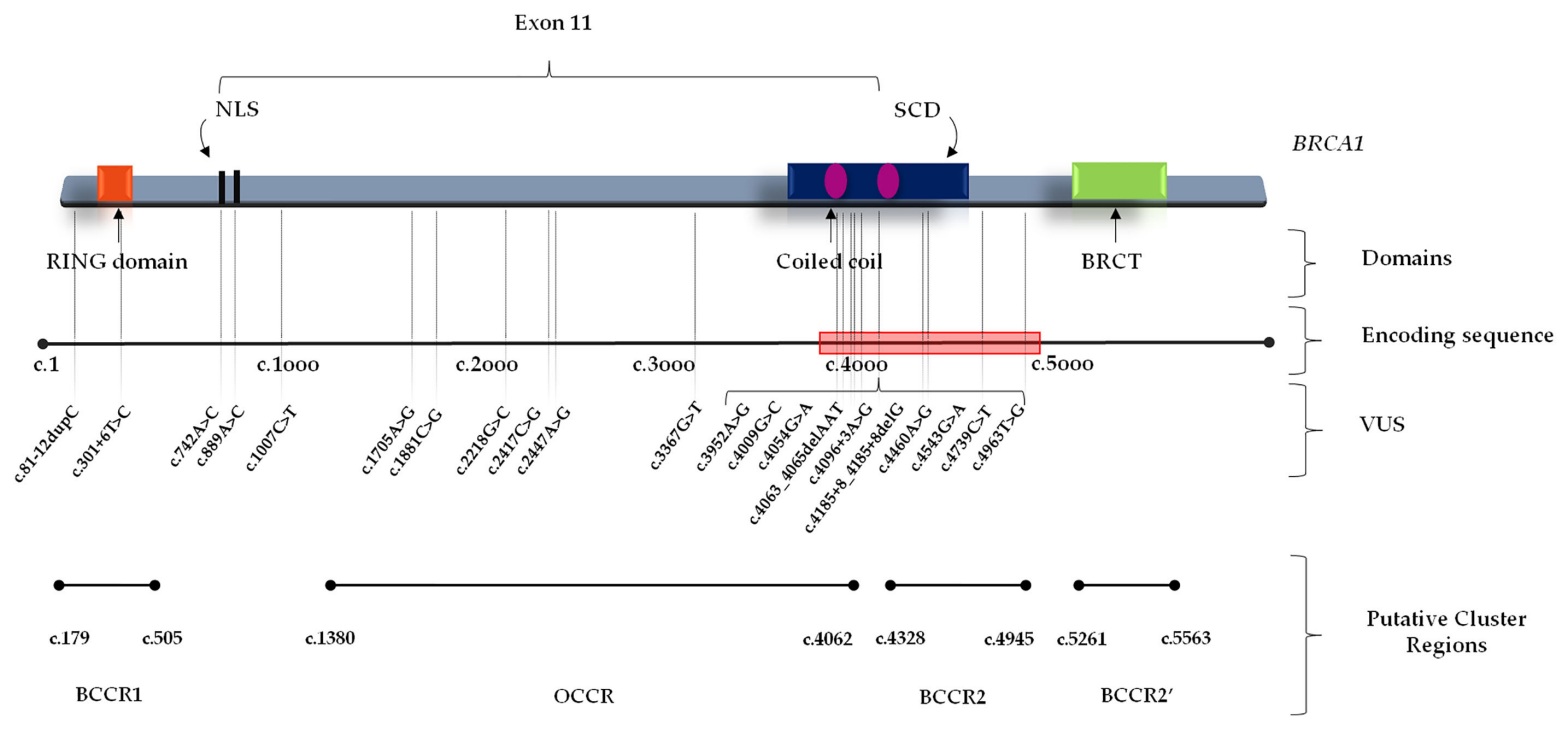
*BRCA1* functional domains and gene location of *BRCA1* Variants of Uncertain Significance in BC and/or OC patients. BCCR, Breast Cancer Cluster Region; BRCT, BRCA1 C-terminus domain; NLS, nuclear localization sequence; OCCR, Ovarian Cancer Cluster Region; SCD, serine cluster domain; VUS, Variant of Uncertain Significance.

Several studies showed a strong correlation between specific *BRCA1/2* variants and changes in BC/OC relative risk, by identifying specific putative Breast Cancer Cluster Regions (BCCRs) and Ovarian Cancer Cluster Regions (OCCRs) located on the coding DNA sequences of *BRCA1/2* genes (30–34). As regards the gene location of *BRCA1* variants detected in our study cohort, most of them (14/21) were mainly located within the hypothetical cluster region present in the BRCA1 protein structure which includes the exon 11 (nucleotides: 861–4,218; codons: 248–1,366), with a greater distribution inside the serine cluster domain (SCD). No variant was observed at BRCT domain near the C-terminus (**Figure 1**). A correlation between the variant localization in the putative BCCRs and OCCRs of *BRCA1* and type of tumor was observed only in three BC and two OC patients (**Table 2**).

**Figure 2:**
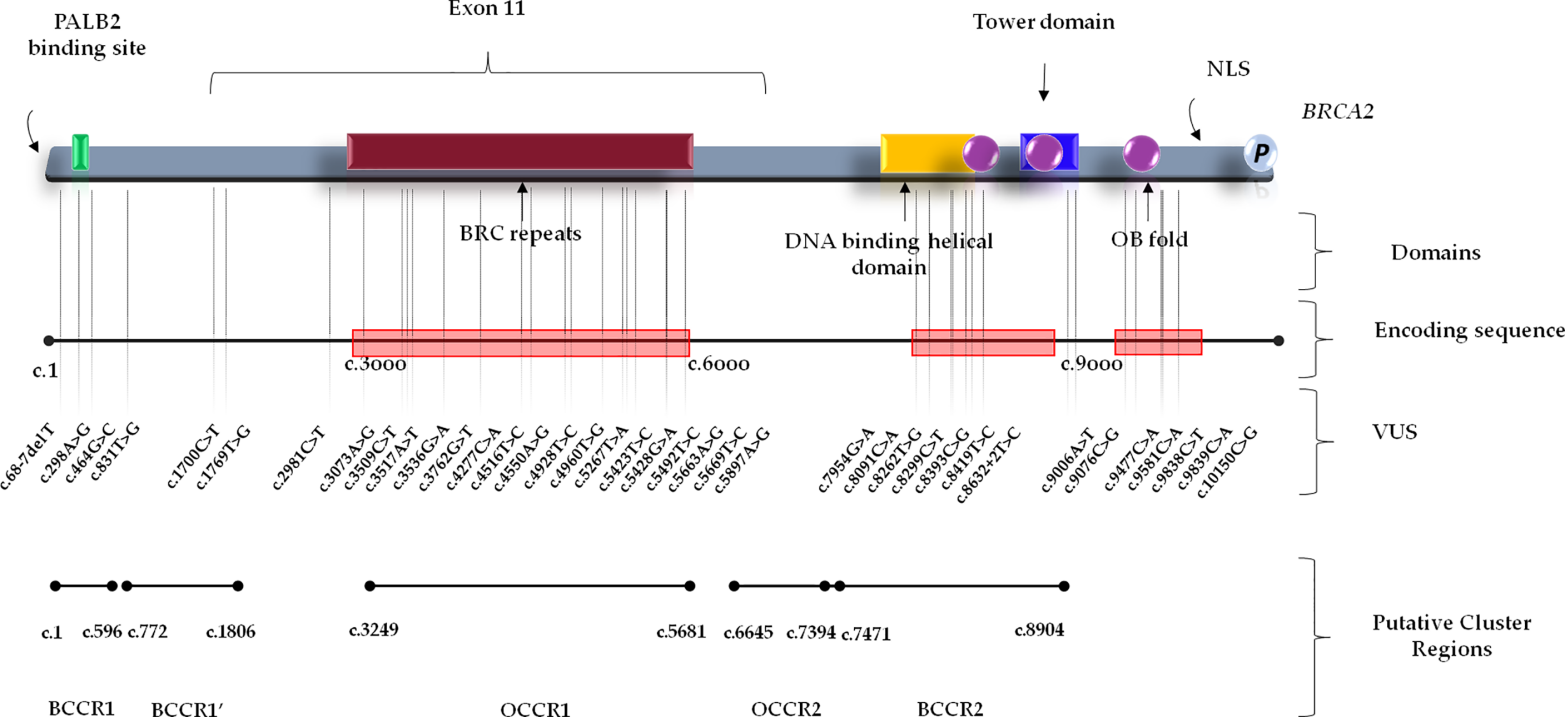
*BRCA2* functional domains and gene location of *BRCA2* Variants of Uncertain Significance in BC and/or OC patients. BCCR, breast cancer cluster region; NLS, nuclear localization sequence; OB, oligonucleotide binding; OCCR, Ovarian Cancer Cluster Region; VUS, Variant of Uncertain Significance.

Conversely, *BRCA2* variants were distributed along the entire gene sequence, but most of them were mainly localized inside three putative cluster regions present in the BRCA2 protein structure which includes the BRC repeats (located within the exon 11), DNA binding helical domain, and OB fold domain near the C-terminus. Specifically, 17 *BRCA2* variants were located at the BRC repeats, included inside exon 11 (nucleotides: 3,301–6,125; codons: 1,025–1,966), whereas seven were detected in the DNA binding helical domain (nucleotides: 8,182–8,862; codons: 2,652–2,878), and, finally, five variants were observed at the C-terminus, within the OB fold domain (nucleotides: 9,705–10,378; codons: 3,159–3,384) (**Figure 2**). A correlation between the variant localization in the BCCRs and OCCRs of *BRCA2* and type of tumor was observed in 16 BC and seven OC patients (**Table 3**).

Online available databases, such as ClinVar, ENIGMA, and VarSome, have been used to perform an *in silico* analysis to investigate the molecular and clinical meaning of an identified variants of unclear significance. Twenty-seven (45.7%) out of 59 observed alterations were defined as VUS in the major databases, whereas 24/59 (40.7%) were categorized as variants in a condition of “Conflicting Interpretations of Pathogenicity” (CIP) on ClinVar database. Furthermore, 3/59 (5.1%) variants have been defined as “not provided” on ClinVar. Among these, the *BRCA1*-c.4963T>G (p.Ser1655Ala) variant identified in three OC women, instead, was reported as Likely Pathogenic on VarSome database. This type of missense variant could be considered disease-causing. Finally, 5/59 (8.5%) variants have been unreported on most common databases and never described until now (**Tables 2** and **3**).

Based on the available information and online databases, after a deep study of personal and familial anamnesis of patients, we described the most representative variants detected in our study cohort.

Interestingly, the co-presence of two different *BRCA2* variants (CIP) named c.9839C>A and c.1769T>G has been observed in three BC probands. The c.9839C>A variant, located in coding exon 26 of the *BRCA2* gene at nucleotide position 9839, has been shown to cause a change in a poorly conserved region of the encoded protein sequence, involving the substitution of a proline amino acid residue with a histidine at codon 3280 (P3280H) (35). Some experimental lines of evidence supported by functional assays reported that this alteration is to be considered as likely benign due to its high frequency in the population and neutral effect on protein function (35, 36), whereas *in silico* tools predicted a damaging effect which could be disease-causing as reported on ClinVar database. For these reasons, the clinical significance of this variant remains yet unclear. The other c.1769T>G alteration causes a nucleotide substitution which involves the replacement of a phenylalanine with a cysteine at codon 590 (F590C) in a known functional domain. Based on current evidence reporting discordant findings, this variant still has an unclear significance (37, 38).

In addition, other two *BRCA2* missense variants, named c.4960T>G and c.4516T>C, have been shown to be simultaneously present in one proband affected by BC. The c.4960T>G alteration involves the change of a thymine with guanine at nucleotide position 4,960 in coding exon 10 of the *BRCA2* gene, resulting in substitution of a cysteine with a glycine at codon 1654, two amino acids with largely different physicochemical properties. The other sequence variant c.4516T>C, instead, determines minor physicochemical changes through the replacement of phenylalanine with leucine at codon 1,506.

Other two sequence variants named c.2447A>G and c.8262T>G (39) located in *BRCA1* and *BRCA2*, respectively, were simultaneously detected in one BC patient. Until today, *in silico* analyses and population frequency data for both alterations have not shown evidence sufficient to associate them with a pathogenicity condition, therefore their clinical significance remains yet unclear.

Furthermore, three OC women have been shown to be carriers of a *BRCA1* sequence variant named c.4963T>G (40), whose interpretation on Clinvar database results to be “not provided”.

**Figure 3:**
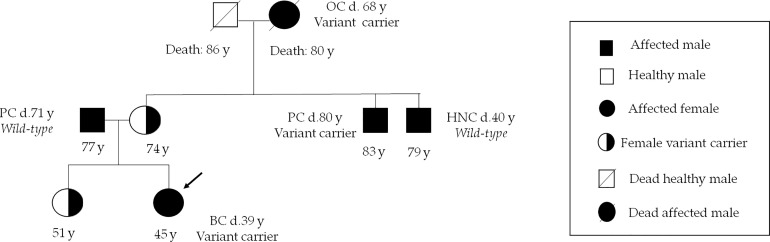
Family pedigree of the c.3367G>T carrier patient. Index case is indicated by an arrow. Numbers under symbols show the age of the patient and relatives. Instead, the age at diagnosis for the affected individuals is indicated by letter “d”. BC, breast cancer; HNC, head and neck cancer; OC, ovarian cancer; PC, prostate cancer.

The *BRCA1* variant named c.3367G>T, which involves the change of aspartic acid with tyrosine (p.Asp1123Tyr) in an unknown functional domain of protein, has been identified in two probands affected by hormone-sensitive BC with early onset (37 and 39 years, respectively). Evidence from some *in silico* studies and low allele frequency observed in large population cohorts supported the hypothesis that this alteration is deleterious (41). Based on currently available data, this alteration is considered to have an unclear clinical significance. However, the case of one of two BC women who were carriers of this variant is interesting. Her family members were offered genetic testing because of the strong family history of *BRCA*-related tumors. Our genetic investigation showed that maternal uncle and grandmother, affected by prostate cancer and OC respectively, were carriers of same variant, whereas patient’s mother and sister were unaffected carriers (**Figure 3**).

Finally, interestingly, 10 variants of unclear significance have been observed in patients who already harbored a *BRCA1* or *BRCA2* PV (**Supplementary Table 1**).

### Description and *In Silico* Analysis of Variants of Unclear Clinical Significance

Though most of the above described variants are known, we detected six alterations in *BRCA1* and two in *BRCA2*, whose interpretation of clinical significance has not reported from data published in literature or main reference databases (**Tables 2** and **3**). We have defined as “not found” the variants not reported on the Clinvar, while those reported in the same database but without an interpretation of clinical significance have been defined as “not provided”.

Three variants, one of which was in *BRCA1* (c.4963T>G) and two in *BRCA2* (c.3536G>A and c.8632+2T>C), were reported as “not provided” on the Clinvar database.

Controversial is the case of *BRCA1* variant named c.4963T>G (p.Ser1655Ala), which has been identified in three OC women. This alteration is defined as “not provided” on Clinvar, whereas it is reported as LPV on VarSome database. Literature data reported that the BRCT domain of BRCA1 is a key binding domain for phosphorylated serine (pSer) (42). Batenburg et al. (43) assessed if S1655 residue might mediate the interaction between BRCA1 and CSB (Cockayne syndrome complementation group B protein), a member of the SWI2/SNF2 superfamily, promoting a DNA repair mechanism *via* homologous recombination. These findings demonstrated that S1655A abolishes the interaction of BRCA1–BRCT with CSB, suggesting that the BRCA1–BRCT complex binds probably to pSer on CSB (43).

The *BRCA2* variant named c.3536G>A (p.Ser1179Asn) has been identified in one 69 years old OC woman. The substitution of a serine with an asparagine at position 1,179 could be considered a novel alteration. The serine residue is located within the BRCA2 region involved in the interaction with RAD51 protein (44, 45). Both asparagine and serine are hydrophilic amino acids and have similar size, but the significance of this alteration is controversial. This *BRCA2* variant of unclear significance has been simultaneously detected in the same patient together with *BRCA1* PV named c.514delC.

The *BRCA2* variant named c.8632+2T>C has been detected in a hormone-sensitive BC patient. This is an intronic variant localized in the splicing regions (46). This variant is actually classified as PV on VarSome, but it was not found on Clinvar. Its meaning is still not well defined.

Three *BRCA1* variants named c.2417C>G and c.81-12dupC, never described before, and c.4185+8_4185+8delG, unreported on ClinVar, but described as VUS on VarSome, have been identified in three out of 46 BC patients, respectively.

Our genetic investigation has allowed identifying two novel frameshift variants, c.81-12dupC and c.4185+8_4185+8delG. Literature data showed that the small frameshift deletion c.4185+8_4185+8delG may lead to a possible alteration damaging the BRCA1 protein (47).

Furthermore, we have detected, in two women respectively, two germline *BRCA1* variants, named c.4543G>A and c.1007C>T, never described before and never reported both on ClinVar and VarSome databases. The c.1007C>T alteration has been considered as “damaging” on SIFT database (48) and has been simultaneously detected in the same patient together with the *BRCA1* PV named c.984_985insC. Further analysis could clarify, in future, the role of this variant.

## Discussion

The frequency of identified VUS worldwide is different and strongly depends on the number of performed genetic testing as well as ancestral origin of the examined population. Literature data reported that VUS prevalence rate reaches 21% for patients of African-American ethnicity, and approximately the 5–6% for individuals of European ancestry (49, 50).

Recently, some studies showed how the prevalence of germline genetic variants and gene-specific risk estimates could change based on family history of cancer or tumor molecular subtype, but also according to other factors such as race, ancestry, and geographic location (51–53).

About 1,500 VUS as well as numerous *BRCA1/2* PVs/LPVs are available in several publicly accessible databases. However, no public databases currently give the possibility to annotate cumulative evidence finalized to reclassify VUS (54).

To understand the clinical meaning of currently identified VUS may be accelerated by enabling increased sharing of information, which results complicated ethical issues (19).

Gene expression studies and *in silico* analysis predicting the impact of the amino acid change on protein folding, by testing the effect of a VUS on functions of a protein have been performed (55, 56).

In our retrospective study, we showed that prevalence of *BRCA1/2* VUS in our population cohort was 7.7%, a concordant value with data reported in literature (15). Some VUS have been shown to be simultaneously present in some enrolled BC and/or OC patients, whereas other variants without an interpretation of clinical significance have been reported and described for the first time.

The collected data about familial anamnesis of a patient harboring the *BRCA1*-c.3367G>T variant suggested that this alteration should be further considered carefully because other affected relatives have been shown to be carriers of this variant.

Our results showed that there is a high heterogeneity of VUS among individuals of our population cohort and only a very few variants are shared between BC or OC patients. The lack of a specific territorial prevalence and heterogeneous distribution of VUS could be attributed to the genetic heterogeneity of the people belonging to regions of Southern Italy and their historical background due to the coexistence of different civilizations and critical geographical position of Sicily at the centre of Mediterranean Sea, crossroads of several ethnicities (57).

This study, through the use of prediction tools and databases, was aimed at describing the *BRCA1/2* variants of unclear significance detected in the Sicilian population, which could be useful, in future, for improving the identification of novel disease-causing variants in *BRCA1* and *BRCA2* genes, allowing their eventual re-classification in potentially high-risk *BRCA* variants eligible for clinical purposes.

To understand whether these variants may potentially belong to class IV/V, an accurate assessment of the proband’s family history should be carried out, offering genetic testing to all consenting family members. However, many variants often cannot be investigated within a family due to the poor knowledge about cancer family history by proband.

In general, the classification of unclear significance variants results to be difficult due to the lack of functional evidence, insufficiency of population-based statistical evidence, and different evaluation approaches by scientists and clinicians (14). Since VUSs are mostly synonymous or missense alterations which involve the substitution of amino acid residues with similar physicochemical properties, or *in-frame* insertions/deletions, their effect on protein function is more complicated to unveil than nonsense variants (58, 14). Therefore, further experimental evidence is requested for enhancing the number of *in vitro* assays, given their complexity and consequent shortage. In addition, in some cases, suitable statistical assessments may be hindered by presence of slightly more frequent variants detected in population subsets or several pathological conditions (18). Finally, if, on one hand, the researchers take into consideration the VUS, polymorphisms, and novel variants because of their potential impact on the biochemical processes (14), even without clinical purposes, on the other hand, instead, the medical geneticists prefer to assess only variants with a well-defined clinical significance which may be helpful in the patient’s clinical management. These different approaches may hamper the studies regarding the clinical interpretation of variants with unclear significance, causing loss of information potentially useful for patient care (59). Another issue is represented by lack of universal standardization method for VUS among different diagnostic laboratories (60).

Nowadays, further *in vitro* functional and *in silico* analysis based on the use of updated databases and predictive algorithms are needed to allow a reclassification of alterations of unclear clinical significance in potentially high-risk variants (58). For this reason, today the development of *in vitro* assays to improve the VUS classification is the main objective of clinical research.

Surely, advances in molecular biology, such as the use of multi-gene panels, exome sequencing, and/or RNA-seq, are increasing the amount of data in the field of research on these unknown variants with unclear significance (61).

Further linkage analyses will be able to provide additional information helpful to geneticists for the understanding of inherited variants of unclear clinical significance associated with BC and/or OC.

## Data Availability Statement

The datasets presented in this article are not readily available because of ethical and privacy reasons. Requests to access the datasets should be directed to antonio.russo@usa.net and viviana.bazan@unipa.it.

## Ethics Statement

The studies involving human participants were reviewed and approved by the ethical committee (Comitato Etico Palermo 1; approval number: 0103-2017) of the University-affiliated Hospital A.O.U.P. ‘P. Giaccone’ of Palermo. The patients/participants provided their written informed consent to participate in this study.

## Author Contributions

Conceptualization: DF, AF, LI, GB, AR, and VB; genetic counseling: DF, VC, CF, AD, RS, and LM; sample collection and gene testing: AF, MB, DC, and AP; data curation and analysis: DF, AF, CB, CF, AD, GM, AC, EP, and LC; writing: DF and AF; Project administration: AR, GB, and VB. All authors contributed to the article and approved the submitted version.

## Funding

This research did not receive any specific grant from funding agencies in the public, commercial, or not-for-profit sectors.

## Conflict of Interest

The authors declare that the research was conducted in the absence of any commercial or financial relationships that could be construed as a potential conflict of interest.

## Supplementary Material

Supplementary Figure 1 Number of genetically tested patients with BC or OC (Oct 2016–Dec 2020). Total number of analyzed patients is divided into carriers of *BRCA* w.t. (green), *BRCA* VUS (yellow), and *BRCA* PVs/LPVs (red). Patients harboring benign/likely benign variants (BVs/LBVs) are considered carriers of *BRCA w*.*t*.
