# Erratum: Prevalence and Spectrum of Germline *BRCA1* and *BRCA2* Variants of Uncertain Significance in Breast/Ovarian Cancer: Mysterious Signals From the Genome

**DOI:** 10.3389/fonc.2022.920342

**Published:** 2022-05-04

**Authors:** Frontiers Production Office

**Affiliations:** Frontiers Media SA, Lausanne, Switzerland

**Keywords:** *BRCA1*, *BRCA2*, breast cancer, genetic testing, ovarian cancer, variants of uncertain significance (VUS)

Due to a production error, there was a mistake in **Table 2** as published. Some *BRCA1* variants were incorrectly listed. The correct **Table 2** appears below. The publisher apologizes for this mistake.

**Table 2:** *BRCA1* gene variants of unclear significance harbored by patients with breast and ovarian cancers.

BRCA1 VUS										
Nucleotide change HGVS nomenclature	Amino acid change	Type of VUS	ClinVar classification	ENIGMA/VarSome	PolyPhen-2/SIFT	HCI Prior/Align-GVGD^a^	BC patients	OC patients	ExAC/GnomAD^b^	pCR
c.3367G>T	p.Asp1123Tyr	MISSENSE	CIP	NYR/VUS	Light/ Damaging	0.02/C0	2	\	0.00002/ 0.00001	OCCR
c.889A>C	p.Met297Leu	MISSENSE	CIP	NYR/VUS	Light/ Tolerated	0.02/C0	1	\	\	\
c.2417C>G	p.Ala806Gly	\	NF	No data	Light/-	0.02/C0	1	\	\	OCCR
c.81-12dupC	\	IVS	NF	No data	\	\	1	\	\	\
c.301+6T>C	\	IVS	VUS	NYR/VUS	\	0.34/\	1	\	0.00002/ 0.00001	BCCR1
c.4063_4065delAAT	p.Asn1355del	In-frame DEL	CIP	NYR/VUS	\	\	1	\	\	\
c.4460A>G	p.Lys1487Arg	MISSENSE	VUS	NYR/VUS	Light/ Tolerated-Damaging	0.02/C0	1	\	\	BCCR2
c.1881C>G	p.Val627=	synonymous	CIP	NYR/LBV	\	0.02/\	1	1	-/0.00003	OCCR
c.2447A>G*	p.His816Arg	MISSENSE	CIP	NYR/VUS	Light/ Tolerated	0.02/C0	1	\	0.00001/ 0.00002	OCCR
c.3952A>G	p.Ile1318Val	MISSENSE	VUS	NYR/VUS	Light/ Tolerated	0.02/C0	1	\	\	OCCR
c.742A>C	p.Thr248Pro	MISSENSE	CIP	NYR/VUS	Light/ Tolerated	0.02/C0	1	\	\	\
c.4185+8_4185+8delG	\	IVS	NF	NF/VUS	\	\	1	\	\	\
c.4054G>A	p.Glu1352Lys	MISSENSE	VUS	NYR/VUS	Light/ Damaging	0.02/C0	1	\	0.00004/ 0.00002	OCCR
c.4739C>T	p.Ser1580Phe	MISSENSE	VUS	NYR/VUS	Light/ Damaging	0.02/C15	1	\	\	BCCR2
c.4009G>C	p.Asp1337His	MISSENSE	VUS	NYR/VUS	Light/ Tolerated	0.02/C0	1	\	\	OCCR
c.2218G>C	p.Val740Leu	MISSENSE	VUS	NYR/VUS	Light/ Tolerated	0.02/C0	1	\	\	OCCR
c.4096+3A>G	\	IVS	VUS	VUS/LPV	\	0.97/\	1	1	\	\
c.4963T>G	p.Ser1655Ala	MISSENSE	Not provided	NYR/LPV	Light/ Damaging	0.03/C0	\	3	\	\
c.4543G>A	p.Gly1515Arg	\	NF	NF/VUS	Light/ Tolerated	0.02/C0	\	1	\	BCCR2
c.1007C>T	p.Thr336Ile	\	NF	NF/VUS	Light/ Damaging	0.02/C0	\	1	\	\
c.1705A>G	p.Asn569Asp	MISSENSE	VUS	NYR/VUS	Light/ Tolerated	0.02/C0	\	1	\	OCCR

*This BRCA1 variant is simultaneously present together with the BRCA2 VUS c.8262T>G (reported in Table 3) in one of probands with BC. Novel variants are reported in bold.

a The Align-GVGD program predicts where the variants in BRCA1 and BRCA2 genes fall in a spectrum ranging from enriched deleterious to enriched neutral. The prediction classes form a spectrum (C0, C15, C25, C35, C45, C55, C65) with C65 most likely to interfere with protein function and C0 least likely. The HCI Prior database, based on Align-GVGD scores, defines four classes of probability of pathogenicity: C0 = 0.03; C15-C25 = 0.29; C35-C55 = 0.66; C65 = 0.81.

b The Exome Aggregation Consortium (ExAC) and Genome Aggregation Database (gnomAD) aggregate both exome and genome sequencing data from a wide variety of large-scale sequencing projects, by providing values of allelic frequency.

BC, breast cancer; BCCR, breast cancer cluster region; CIP, conflicting interpretations of pathogenicity; DEL, deletion; HGVS, Human Genome Variant Society; IVS, intronic variants; LBV, likely benign variant; LPV, likely pathogenic variant; NF, not found; NYR, not yet reviewed; OC, ovarian cancer; OCCR, ovarian cancer cluster region; pCR, putative cluster region (defined by Rebbeck et al.); VUS, variant of uncertain significance.

The original version of this article has been updated.

