# Supplementary figures and images for: Prevalence and Spectrum of Germline BRCA1 and BRCA2 Variants of Uncertain Significance in Breast/Ovarian Cancer: Mysterious Signals From the Genome
Source: Front Oncol. 2021 Jun 11;11:682445. doi: 10.3389/fonc.2021.682445 (PMC8226162; doi:10.3389/fonc.2021.682445)

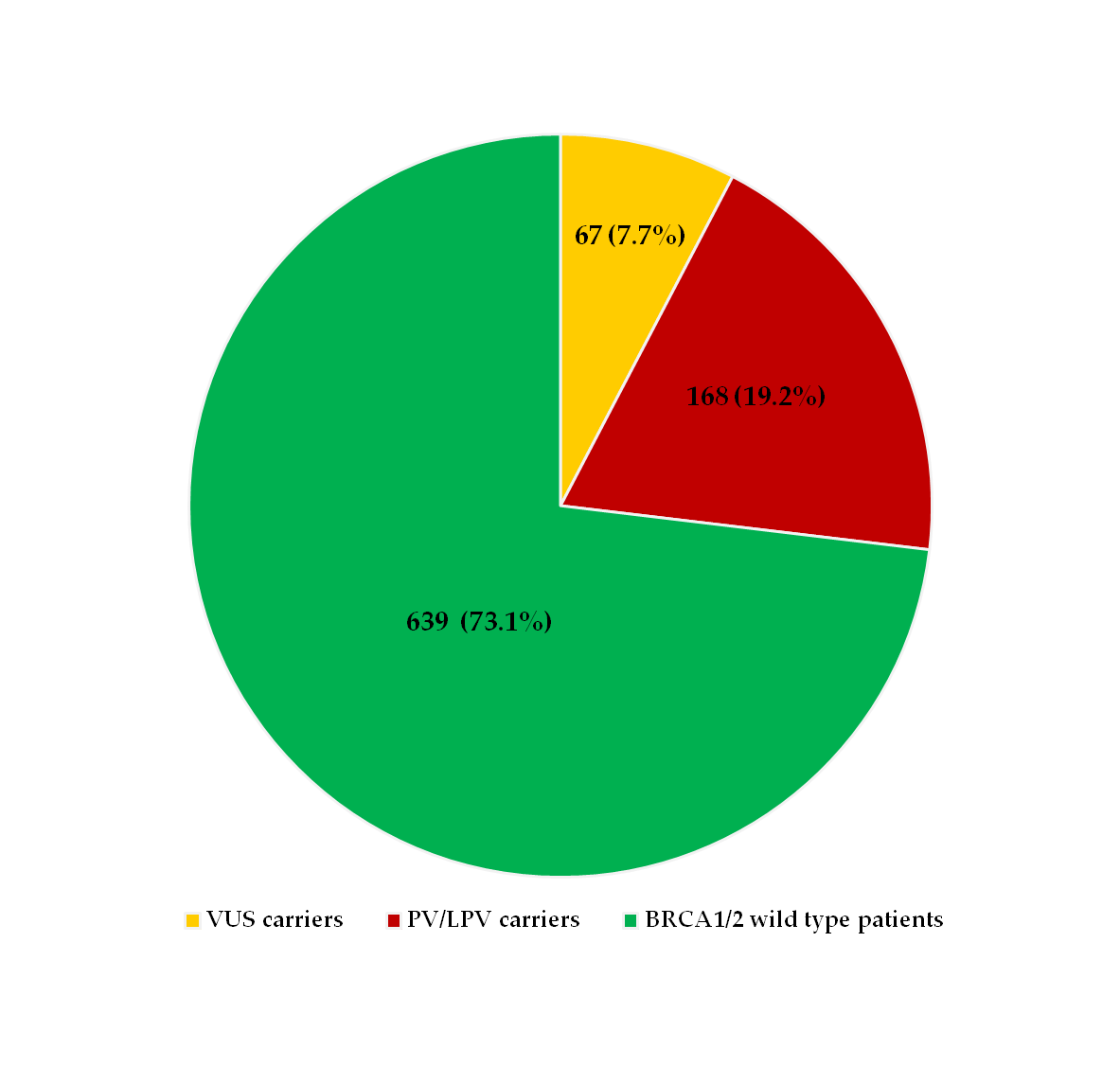

Supplement: Supplementary Figure 1 — Number of genetically tested patients with BC or OC (Oct 2016–Dec 2020). Total number of analyzed patients is divided into carriers of BRCA w.t. (green), BRCA VUS (yellow), and BRCA PVs/LPVs (red). Patients harboring benign/likely benign variants (BVs/LBVs) are considered carriers of BRCA w.t. [file Image_1.tif]
