# Supplementary material for: Prevalence and Spectrum of Germline BRCA1 and BRCA2 Variants of Uncertain Significance in Breast/Ovarian Cancer: Mysterious Signals From the Genome
Source: Front Oncol. 2021 Jun 11;11:682445. doi: 10.3389/fonc.2021.682445 (PMC8226162; doi:10.3389/fonc.2021.682445)
Supplement: Supplementary file 2 [file Table_1.docx]

**Supplementary Table 1. Variants of unclear significance simultaneously detected in carriers of *BRCA1* or *BRCA2* PVs**

| **Carriers of PV and VUS** | | | | | | |
| --- | --- | --- | --- | --- | --- | --- |
| **ID Patient** | **VUS - HGVS nomenclature** | **Protein change** | **Type of variant** | **Gene** | **PV - HGVS nomenclature** | **Gene** |
| **BC06** | c.301+6T>C | \ | [VUS](https://www.ncbi.nlm.nih.gov/clinvar/variation/224562/) | *BRCA1* | c.1238delT | *BRCA2* |
| **OC15** | c.4550A>G | p.Lys1517Arg | [VUS](https://www.ncbi.nlm.nih.gov/clinvar/variation/141066/) | *BRCA2* | c.181T>G | *BRCA1* |
| **OC16** | c.3536G>A | p.Ser1179Asn | [Not provided](https://www.ncbi.nlm.nih.gov/clinvar/variation/51483/) | *BRCA2* | c.514delC | *BRCA1* |
| **BC26** | c.3517A>T | p.Ile1173Phe | [VUS](https://www.ncbi.nlm.nih.gov/clinvar/variation/96793/) | *BRCA2* | c.514delC | *BRCA1* |
| **OC09** | c.3517A>T | p.Ile1173Phe | [VUS](https://www.ncbi.nlm.nih.gov/clinvar/variation/96793/) | *BRCA2* | c.4964_4982del19 | *BRCA1* |
| **BC09** | c.1881C>G | p.Val627= | [CIP](https://www.ncbi.nlm.nih.gov/clinvar/variation/54378/) | *BRCA1* | c.7007G>A | *BRCA2* |
| **OC18** | c.5492T>C | p.Ile1831Thr | [VUS](https://www.ncbi.nlm.nih.gov/clinvar/variation/141782/) | *BRCA2* | c.4964_4982del19 | *BRCA1* |
| **BC12** | c.742A>C | p.Thr248Pro | [CIP](https://www.ncbi.nlm.nih.gov/clinvar/variation/252390/) | *BRCA1* | c.5266_5267insC | *BRCA1* |
| **OC20** | c.5669T>C | p.Met1890Thr | [VUS](https://www.ncbi.nlm.nih.gov/clinvar/variation/51907/) | *BRCA2* | c.547+2T>A | *BRCA1* |
| **OC07** | c.1007C>T | p.Thr336Ile | NF | *BRCA1* | c.984_985insC | *BRCA1* |

Abbreviations: CIP, Conflicting Interpretations of Pathogenicity; HGVS, Human Genome Variant Society; NF, Not Found;

PV, Pathogenic Variant; VUS, variant of uncertain significance.
